# Embayed beach configuration explained by wave sheltering

**DOI:** 10.1038/s41598-024-51574-x

**Published:** 2024-01-11

**Authors:** Ana Nobre Silva, Rui Taborda, César Andrade

**Affiliations:** Departamento de Geologia, Instituto Dom Luiz, Faculdade de Ciências da Universidade de Lisboa, Edifício C6, Campo Grande, 1749-016 Lisbon, Portugal

**Keywords:** Physical oceanography, Ocean sciences, Geomorphology

## Abstract

Embayed beaches, characterized by their distinctive planform curvature, are a common feature along coastlines worldwide. This study introduces a novel approach to describe bay shape that extends our understanding about the processes that control embayed beach development. The novel approach was thoroughly tested against one-line models and applied to real-world cases covering a wide range of spatial scales, wave climate conditions and geomorphological settings. Here we show that the equilibrium curvature of embayed beaches can be correctly described and explained by considering headland-provided offshore wave sheltering alone, without explicitly accounting for wave shoaling, refraction, diffraction, or longshore transport. This holds true as long as the offshore wave climate is accurately characterized, including complete information regarding the mean and the spread of the incoming wave direction. For narrow-banded dominant swell wave regimes, the inclusion of background wind sea components has been identified as crucial for predicting curvature in the more sheltered embayed domain. The presented model significantly contributes to the understanding of how waves shape embayed beaches.

## Introduction

Beaches hold significant socioeconomic value and provide essential ecosystem services^1,2^. They are dynamic environments, continuously changing their shape in an endless effort to adapt to the oceanographic forcing^3^. Recognizing the controls on beach planform contributes to the understanding of present-day beach dynamics and provides valuable insights into how beaches will respond to future changes in forcing due to climate change.

Embayed beaches are a type of beaches that develop along rocky coasts and comprise about half of the world’s coasts^4,5^. They are characterized by a distinctive curved planform and develop in relation to protruding headlands. The degree of shoreline curvature mirrors the search for equilibrium between sediment supply and wave-driven distribution processes^6–8^, which are highly sensitive to natural and anthropogenic-induced disturbances. Embayed beaches can exist in either static or dynamic equilibrium states^9^. Static equilibrium occurs when predominant waves arrive normal to the beach and break simultaneously along the entire curved shoreline, resulting in negligible net longshore sediment transport. In contrast, dynamic equilibrium is maintained when sediment is supplied to the beach from updrift or within the embayment and exits at an equal rate. This leads to a less indented shoreline that develops seaward of the static equilibrium configuration. Both equilibrium states assume long-term planform stability and fall under the broader category of equilibrium embayed beaches, a term adopted in this study.

The planform of embayed beaches, originally described by Silvester^10^ as half-heart shaped, has been studied using various modelling approaches, including empirical, process-based, data-driven, and hybrid models^5,7,11–13^. Among these, empirical and hybrid models are the most commonly utilized.

Empirical models use predefined curves, such as logarithmic spirals^14,15^, parabolic bay shapes^16–22^ and hyperbolic tangent shapes^11,23^. The real-world application of these models depends on the assessment of several empirical fitting parameters, including scaling and location parameters that are not directly linked to the physical processes that drive bay configuration and evolution^24^. Additionally, some assumptions made by these models, such as the use of a single wave to represent the entire wave climate, are often not met in nature.

Hybrid approaches, also called one-line shoreline models in ref.^5^, couple oceanographic forcing with the morphological response of the beach^5,24–28^. While these approaches vary in their level of simplification and the physical processes they describe, they generally: (i) resolve the propagation of deep-water waves to the shore; (ii) use the nearshore wave regime to estimate potential sediment transport rates; and (iii) compute shoreline planform using transport rates and the continuity equation. For instance, the research conducted by ref.^24^ proposes a one-line model that integrates wave-induced sediment transport processes to simulate the long-term changes in crenulate bay shorelines. This model was used to investigate the sensitivities of crenulate bay shorelines concerning dominant wave direction and wave spreading. Their findings suggest that one-line models can be useful tools for predicting shoreline evolution and the equilibrium configuration of embayed beaches.

However, the successful application of one-line models requires significant effort and expertise including gathering the necessary data to initialize and calibrate the model (e.g., bathymetric data, shoreline data, wave data, tidal data, sediment characteristics) and model configuration (e.g., setting up the computational grid, specifying boundary conditions, defining initial conditions, and selecting appropriate parameter values). Moreover, model applications require a deep understanding of the physics and processes governing coastal morphodynamics and, to ensure their accuracy and reliability, they must be calibrated and validated against observed data. These characteristics have restrained their ready application to real beach cases worldwide. In practice, one-line models have been applied primarily to synthetic cases and, in most cases, to a single real-case embayment. To overcome these limitations, it is desirable to develop “appropriate complexity” models that balance reductionism and synthetism to capture the behaviours necessary for explanatory and predictive capabilities^29^.

The objective of the current research is to establish a model that helps to understand the fundamental mechanisms underlying the planform development of equilibrium embayed beaches and provides predictive capabilities at the mesoscale. Here we investigate the hypothesis that beach curvature is directly related to the line-of-sight of offshore waves, which means that beach planform can be estimated considering solely offshore wave sheltering without the need for nearshore wave transformation or longshore sediment transport computations.

The novel reduced-complexity Beach Planform Model (BPM) presented in this work offers a simplified and efficient approach for predicting embayed beach equilibrium planform. To outline briefly, the BPM iteratively builds the equilibrium planform of beaches by assessing variations in sheltering provided by headlands, along the embayment. The iterative process initiates at the downwave beach limit (beach start) by computing the mean direction of the in-line-of-sight offshore waves. A shoreline segment perpendicular to that direction is then added to the beach, and the iterative process continues until reaching the upwave beach end (see the detailed BPM approach in Methods; Extended Data Figs. 1–2). The ability of the BPM to reproduce shoreline curvature was assessed using both synthetic, or idealized, scenarios as well as real-world cases representing various spatial scales, geomorphological settings, and wave climates.

## Idealized beach scenarios

In a series of idealized wave climate scenarios, Hurst et al.^24^ using a one-line model, demonstrated that the relief of the equilibrium planform bay increases not only with the obliquity of the mean wave direction (ϴmean) but also decreases with the spread of wave directions (ϴstd).

**Figure 1:**
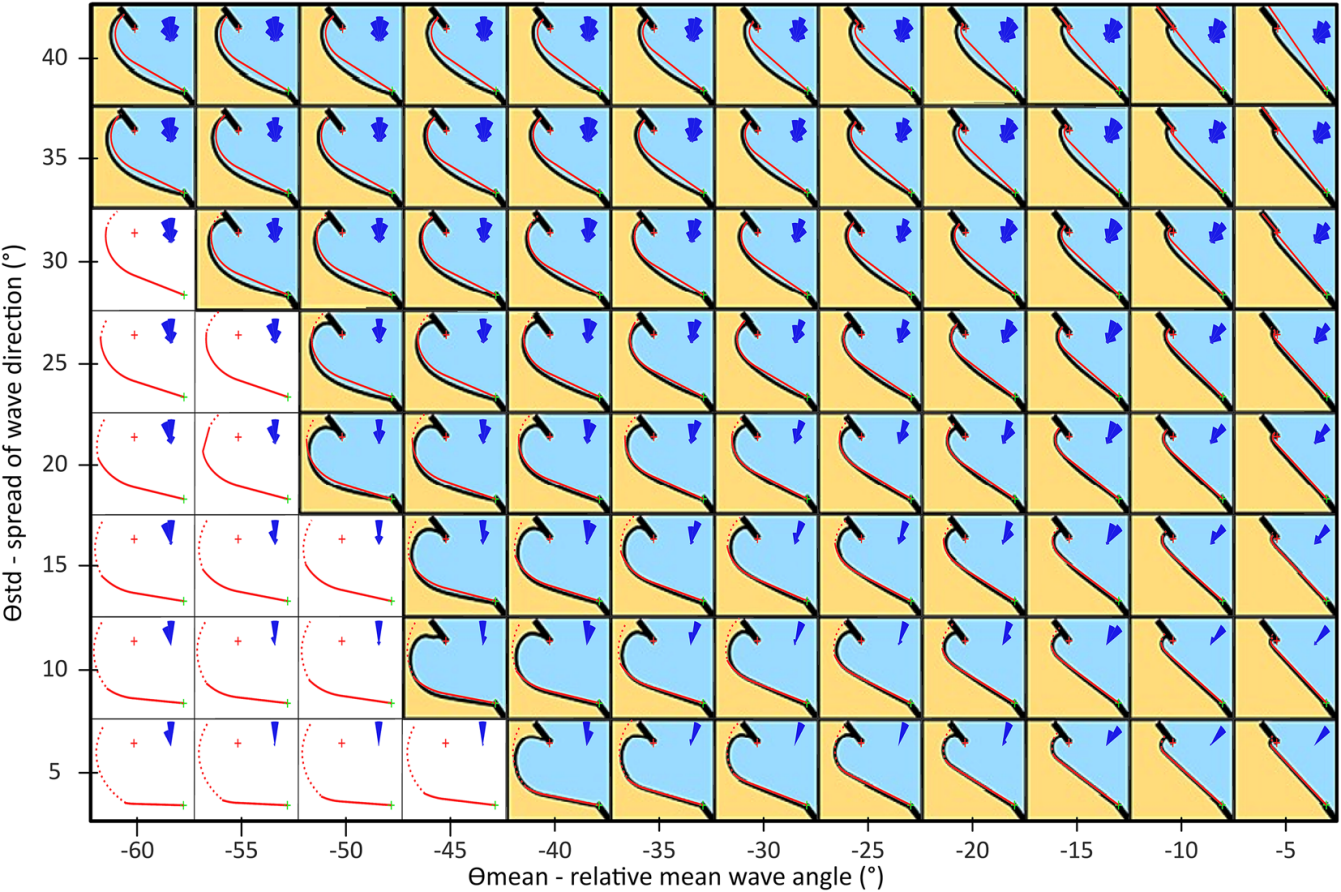
BPM’s simulated shorelines as a function of wave direction and spreading overlayed on Hurst et al.^24^ results. The dark blue wave roses in upper-right corners represent the synthetic offshore wave climate; red and green crosses mark the UW and DW fixed-points, respectively. The solid-red lines represent the shorelines produced by BPM, and the dashed-red lines indicate the extended BPM’s solution for shadowed areas, including wide banded sea. The background image is adapted from Hurst et al.^24^ results, shown as black-solid lines, which were obtained using a one-line shoreline model; the white background fields indicate high-angle wave instability (HAWI) conditions.

The embayment configurations produced by the BPM, in the same set of scenarios, show a striking resemblance, in terms of shoreline curvature and orientation, to the outcomes yielded by the aforementioned model^24^. This similarity is observed across an extensive range of relative wave angles (− 60° < ϴ_mean_ < − 5°) and spread of wave directions (5° < ϴ_std_ < 40°) (Fig. 1).

In the more exposed areas of the embayment the shoreline aligns perpendicularly with respect to mean offshore wave direction, regardless of the spread on incident wave direction. Consequently, in instances where the relative wave angle is low (|ϴ_mean_| ≈  < 15°), headlands are connected by a straight beach section with only a slight indentation in its upwave region. On the other hand, high relative angles (|ϴ_mean_| ≈  > 20°) promote increased shoreline curvature with various forms depending on the spread of wave direction.

When the spread of wave direction of the synthetic wave climate is very narrow (corresponding to a low θ_std_ value), the less exposed sector of the beach may become completely sheltered (out-of-sight) from all offshore waves. In the absence of refraction and diffraction, this shadowed area would be entirely devoid of waves, rendering the BPM modelling approach infeasible. However, in real-world scenarios, a small wind wave component is always present, even under very narrow-banded swell conditions (as observed in real beach case applications). To address this limitation in the idealized scenarios simulated by BPM, a background low-energy (less than 1%) wide-banded wind wave was introduced (as described in the Methods). This procedure allows the BPM to accommodate more realistic conditions and ensures that the entire embayed beach is simulated. Simulations with this additional sea component are represented by a dashed-red line, which represents the sectors of the beach that are sea-dominated, while the solid-red line represents the swell-dominated sectors (Fig. 1). The connection between the unsheltered beach, which is characterized by low-curvature and dominated by swell, and the sheltered domain, which is dominated by sea, is made by an acute angle that gradually becomes smoother as the standard deviation of the incident wave direction (θ_std_) increases. The main differences between the model by Hurst et al.^24^ and BPM were observed in the shadowed sectors of the beach. It should be noted that the BPM was able to produce results in the HAWI (high-angle wave instability) zone^24,30^ where the wave spreading is less than 35° and the relative wave direction is higher than 40° (Fig. 1).

## Real beach cases

Real-world embayments exhibit remarkable morphological diversity, shaped by numerous factors each contributing to the uniqueness of each beach. Key factors include the geological setting, oceanographic forcing and sediment supply, which pose challenges to the application of reduced-complexity shoreline models that rely on a limited set of parameters. This is the case of BPM that builds the planform of embayed beaches solely based on the knowledge of wave sheltering by the upwave headland (UW). To address the complexities related to wave propagation effects in the vicinity or within embayments, such as those arising from the presence of bathymetric heights, multiple protruding headlands, or coastal sheltering from islands^31^, we developed a simple yet effective optimization strategy. This strategy seeks to determine the optimal position for the upwave (UW) fixed-point, which represents the effective headland position, for accurately reproducing wave sheltering along the embayment (see Methods and Extended Data Fig. 1 for description of the optimization).

**Figure 2:**
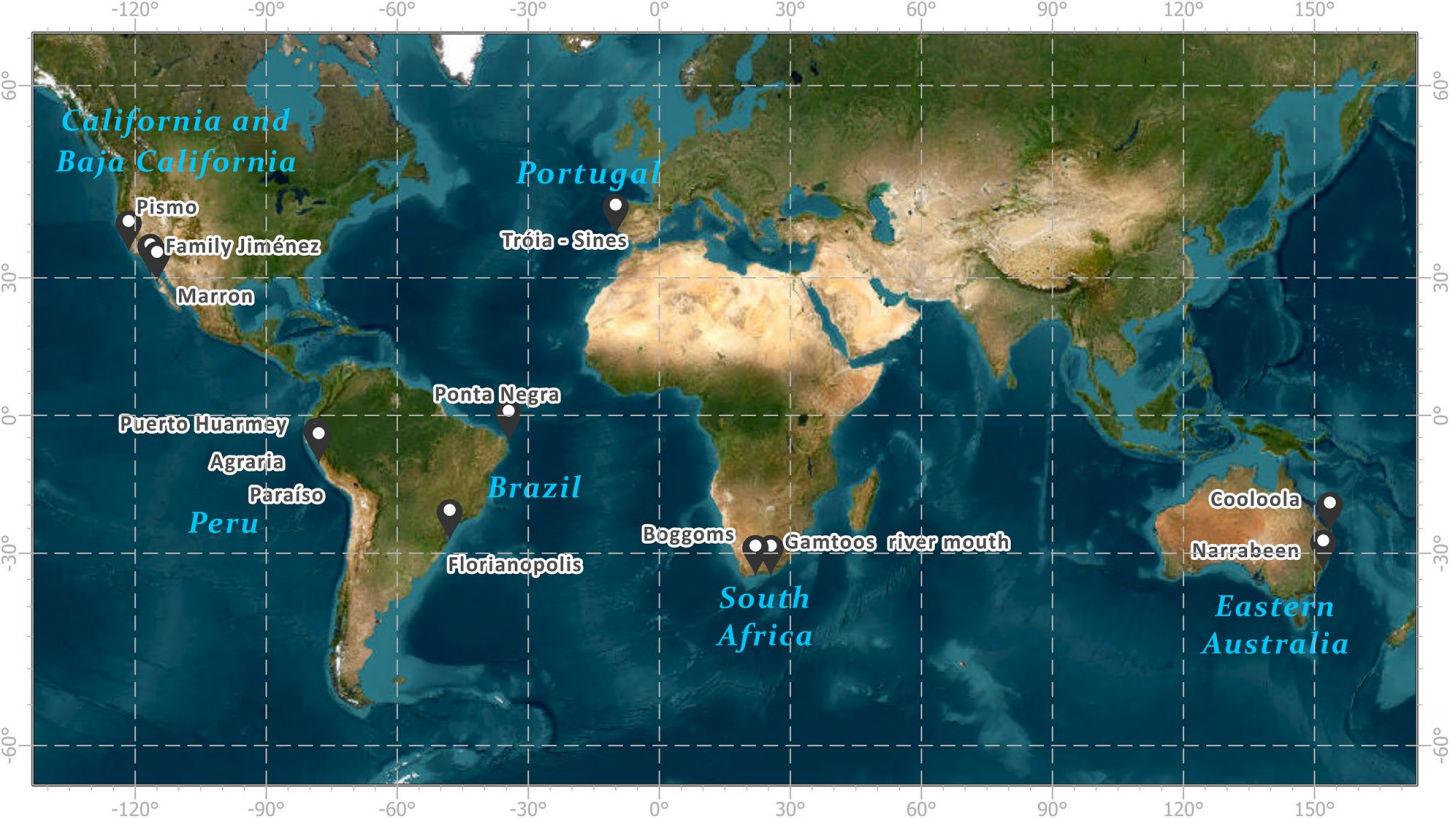
Study site locations. Locations of the 13 embayed beaches worldwide grouped into seven regions: Portugal, Australia, Brazil, South Africa, USA, Mexico and Peru. Map created in ArcGIS Pro 3.2.0, basemap imagery source is Esri’s Earthstar geographic, https://www.esri.com/en-us/arcgis/products/arcgis-pro.

**Figure 3:**
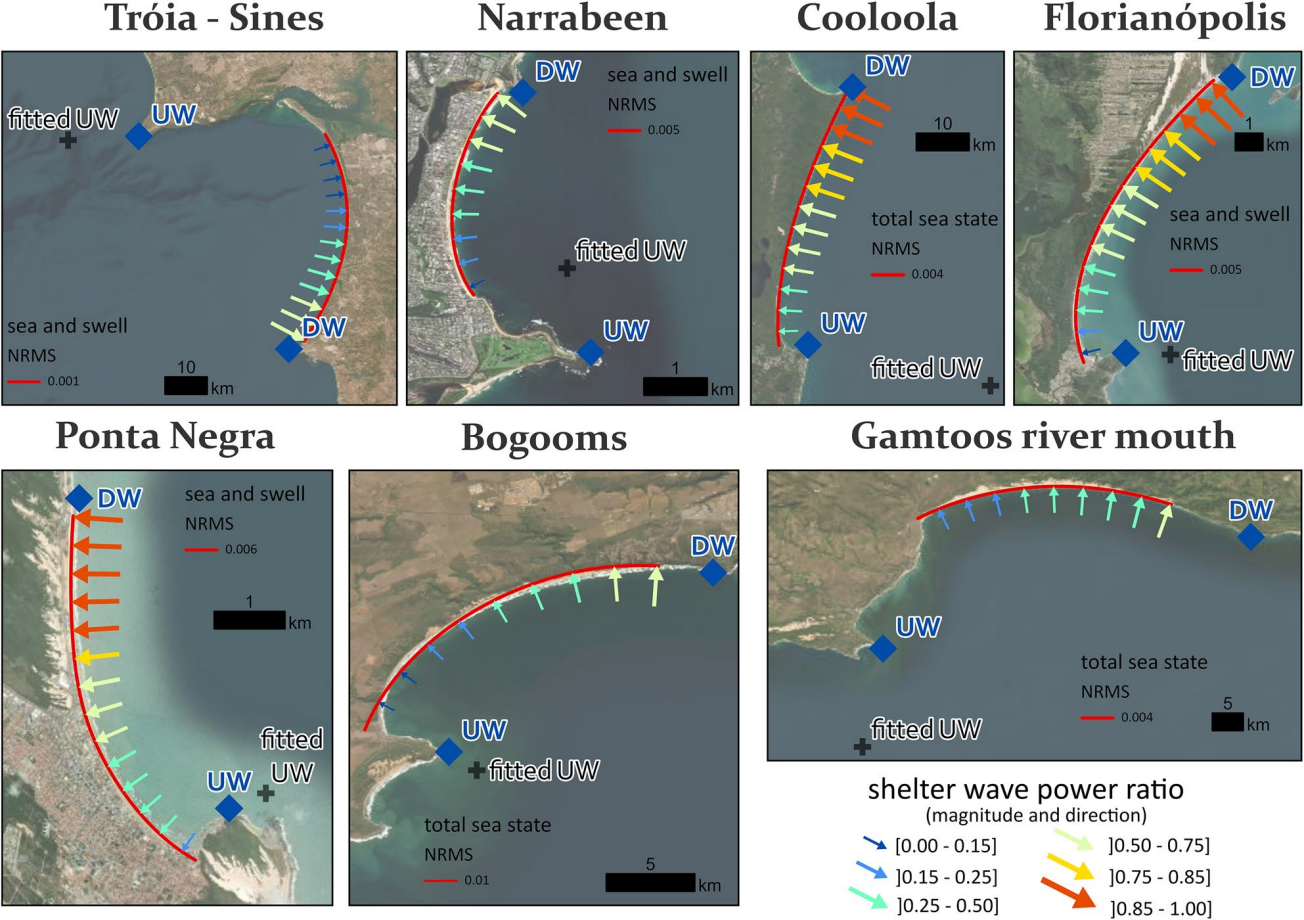
BPM’s optimized shorelines at beach cases in Portugal, Eastern Australia, Brazil and South Africa. The solid-red lines represent the best-fitted shoreline simulation at seven embayed beaches (see Table 1 for Fitting statistics), with the best-fitting criteria being the minimum RMS for either the total sea state or the sea and swell partitions. The blue diamonds indicate the downwave (DW) and upwave (UW) headland positions, while the black crosses represent the optimized (fitted) UW position given by the Optimization Function (OF). The coloured arrows represent the incident (in-line-of-sight) wave climate, with arrow size and colour indicating magnitude, and arrow direction indicating mean wave power direction along the embayment. Map created in ArcGIS Pro 3.2.0, basemap imagery source is Esri’s Earthstar geographic, https://www.esri.com/en-us/arcgis/products/arcgis-pro.

**Figure 4:**
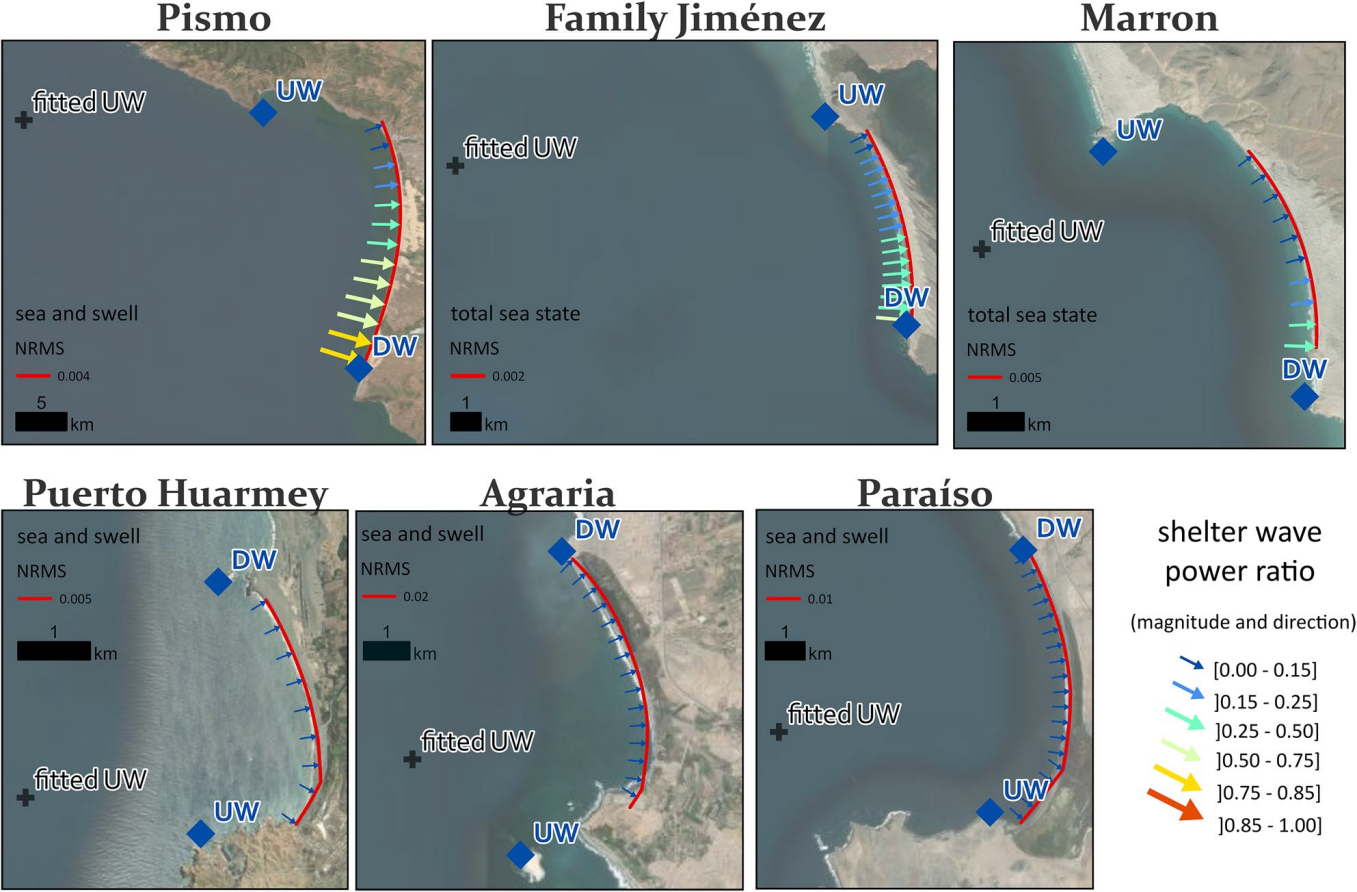
BPM’s optimized shorelines at beach cases in California, Baja California and Peru. The solid-red lines represent the best-fitted shoreline simulation at seven embayed beaches (see Table 1 for Fitting statistics), with the best-fitting criteria being the minimum RMS for either the total sea state or the sea and swell partitions. The blue diamonds indicate the downwave (DW) and upwave (UW) headland positions, while the black crosses represent the optimized (fitted) UW position given by the Optimization Function (OF). The coloured arrows represent the incident (in-line-of-sight) wave climate, with arrow size and colour indicating magnitude, and arrow direction indicating mean wave power direction along the embayment. Map created in ArcGIS Pro 3.2.0, basemap imagery source is Esri’s Earthstar geographic, https://www.esri.com/en-us/arcgis/products/arcgis-pro.

The BPM was applied in 13 beaches worldwide, representing a broad range of spatial scales (3–50 km), mean significant wave height (1.3–2.8 m), directional wave spread (9°–41°), wide-ranging sheltering conditions (from nearly fully sheltered to exposed) and diverse geomorphological settings (Figs. 2, 3, 4, Table 1 and Extended Data Fig. 3). The shoreline simulations were conducted by utilizing only offshore wave climate data, the positions of the headlands (UW and DW), a beach start point and a single optimization parameter. This parameter corresponds to repositioning the UW point, which is initially defined at the land/sea tip of the headland, to a more seaward location (see Methods, fitted UW in Figs. 3, 4 and Head. dist. in Table 1). Using this approach, the model results were found to align very well with the actual shorelines for all cases.

**Table 1:** Summary of oceanographic and geomorphological settings for the 13 studied embayed beaches, and statistics of BPM’s optimized simulations considering total sea state and sea and swell partitions.

	Country		Portugal	Australia		Brazil		South Africa		USA	Mexico		Peru		
	Embayed beach		Tróia -Sines	Narrabeen	Cooloola	Florianópolis	Ponta Negra	Boggoms	Gamtoos river mouth	Pismo	Family Jiménez	Marron	Puerto Huarmey	Agraria	Paraíso
Offshore oceanographic settings	ERA5 offshore wave data position	Lat (°)	38.5	− 33.5	− 26.0	− 27.5	− 6.0	− 34.5	− 34.5	35.0	30.5	29.0	− 10.0	− 11.0	− 11.0
		Long (°)	− 10.0	151.5	153.5	− 48.0	− 34.5	22.0	25.5	− 121.5	− 116.5	− 115.0	− 78.5	− 78.0	− 78.0
	Significant wave height (m)	Average	2.2	1.3	1.5	1.6	1.8	2.2	2.8	2.2	1.7	1.4	1.8	1.8	1.8
	Wave period (s)	Average	9	7	7	8	8	9	10	9	9	9	10	10	10
	Wave direction (˚)	Average	316	127	109	121	99	193	200	293	280	281	201	202	202
	Wave power direction (°)	Average	311	139	117	137	98	200	211	295	284	285	201	202	202
		SD	27.6	40.8	31.6	40.7	34.6	33.3	39.0	18.7	18.8	18.1	8.6	9.0	9.0
Embayment settings	Beach length (m)		50781	3344	50754	11550	5444	17726	35282	24366	6522	3713	3321	5881	7325
	Beach bearing angle (˚)		185	5	13	23	339	55	84	181	167	162	353	348	1
	Distance between beach limits (m)		48917	3184	49758	10819	5002	15898	33617	23718	6415	3635	3117	5150	6797
	Beach curvature		0.96	0.95	0.98	0.94	0.92	0.90	0.95	0.97	0.98	0.98	0.94	0.88	0.93
Simulated shoreline with total sea state	Shelter wave power ratio	Average	0.29	0.37	0.66	0.65	0.52	0.29	0.32	0.32	0.27	0.16	0.0001	0.001	0.00003
		Min	0.06	0.06	0.28	0.21	0.15	0.03	0.19	0.08	0.13	0.04	8.E−06	8.E−06	8.E−06
		Max	0.61	0.59	0.89	0.90	0.92	0.69	0.55	0.70	0.52	0.44	0.0004	0.006	0.0001
		SD	0.17	0.16	0.18	0.20	0.23	0.17	0.10	0.19	0.11	0.11	0.0001	0.001	0.00001
	Fitting stats	Head. dist. (m)	7383	1339	31912	1321	1371	1666	16078	24772	10784	2567	11988	6905	139022
		RMS (m)	58.91	20.06	*188.75*	63.18	57.66	*247.90*	*144.83*	130.84	*10.68*	*18.12*	156.12	245.87	508.89
		NRMS	0.001	0.006	*0.004*	0.005	0.011	*0.014*	*0.004*	0.005	*0.002*	*0.005*	0.047	0.042	0.069
Simulated shoreline with sea and swell partitions	Shelter wave power ratio	Average	0.26	0.37	0.68	0.60	0.62	0.26	0.28	0.44	0.40	0.22	0.001	0.005	0.001
		Min	0.06	0.05	0.25	0.11	0.15	0.09	0.14	0.11	0.22	0.05	1.E−07	3.E−07	3.E−07
		Max	0.60	0.61	0.91	0.88	0.94	0.59	0.54	0.80	0.70	0.59	0.004	0.056	0.004
		SD	0.17	0.18	0.19	0.23	0.25	0.13	0.11	0.23	0.14	0.14	0.001	0.011	0.001
	Fitting stats	Head. dist. (m)	13096	1358	25217	1445	559	4082	18033	19416	11230	2310	2443	3101	5650
		RMS (m)	*58.06*	*17.10*	190.51	*56.17*	*34.60*	271.02	148.77	*88.01*	12.12	19.72	***17.07***	***125.32***	***71.46***
		NRMS	*0.001*	*0.005*	0.004	*0.005*	*0.006*	0.015	0.004	*0.004*	0.002	0.005	***0.005***	***0.021***	***0.010***

The italic cells indicate the best-fitting solutions for each study site, while the bold cells highlight the embayed beaches where the sea and swell partitions significatively improved the model simulations. The beach bearing angle is the azimuth of the line connecting the beach endpoints looking in the downwave direction, and the beach curvature is the ratio of the distance between beach endpoints to the beach length (see “Methods” for additional details).

The applications of BPM to the real-world cases described below illustrate multifaceted dynamics of wave sheltering and serve as valuable examples for exploring and understanding the processes that shape the bays.

The application of BPM to Narraben embayment in Eastern Australia demonstrates how the optimization process resolves ambiguity in selecting an appropriate upwave point location, an issue shared by empirical approaches in the definition of the "diffraction point" ^32^. The strategy employed by BPM to address this ambiguity has resulted in a solution that closely resembles the actual planform of the beach, even in the more sheltered areas of the embayment (Fig. 3 and Extended Data Fig. 4). While there are minor offshore and inshore misfits at the central and northern sections of the embayment, respectively, the global positional RMS error is only about 17 m (Table 1), which is favourable compared to the 24 m RMS error yielded by the LX-Shore processed-based model^33^. The optimization strategy resulted in a fitted UW position displaced about 1.3 km seaward of the visible tip of the headland (Head. dist. in Table 1), which appears to be largely due to the presence of a topographic high^33^, acting as a "bathymetric lens" ^34^ that increases bay sheltering.

Simulating beach curvature in complex geomorphological settings is exemplified in Family Jiménez and Marron beaches in Mexico, where the model successfully reproduces the regional sheltering effects caused by the presence of an island to the northwest (see Family Jiménez in Fig. 4 and Extended Data Fig. 7) and prominent headlands near the beach (see Marron in Fig. 4 and Extended Data Fig. 7). The fitted UW position is shifted seaward by approximately 10 km and 2 km at Family Jiménez and Marron, respectively (Head. dist. in Table 1). Notably, BPM's simulated shorelines in both cases exhibit RMS errors of less than 2 tens of meters, which is less than 0.5% of their respective beach lengths (Table 1).

BPM was also able to reproduce the planform of equilibrium embayed beaches with net longshore sedimentary fluxes along the embayment. The existence of longshore transport is translated in an angular difference (γ_d_) between the direction of the offshore mean wave energy flux and the beach orientation down-coast^20^. While BPM assumptions may seem in contradiction with the need for some wave obliquity required for maintaining net longshore drift, this was resolved by empirically displacing UW seaward, which increases wave sheltering and produces a rotational shift of incident wave energy at the beach to compensate for γ_d_. This strategy has been successfully implemented in cases such as Cooloola and Ponta Negra (Fig. 3, Table 1 and Extended data Figs. 4–5), where BPM simulations have lived up to expectations, with normalized RMS (NRMS) errors of up to 0.6% (Table 1).

Our study examined the influence of wave climate description on BPM outcomes. We found that, in general, using total sea state produced accurate results, although decoupling sea and swell also yielded comparable outcomes (Table 1, Extended Data Figs. 4–8, refer to Methods for computational details). However, there were some exceptions to this trend, specifically in areas with highly oblique offshore swells that are extremely narrow-banded. This is the case of Puerto Huarmey, Agraria, and Paraíso in Peru, which represent the most extreme sheltering conditions investigated in our study. In these embayments, the shelter wave power ratio SWPR (see Methods for details) is always lower than 6%, and their shadowed side (UW side) is fully out-of-sight for swell waves (Extended data Fig. 3). Here, decoupling of total sea state into sea and swell partitions allowed to expose the otherwise shadowed side of the beach to low-energy sea components and thus extend BPM application to the entire shoreline. The successful application of this strategy is well illustrated in Paraiso (Extended Data Fig. 8). Specifically accounting for the sea partitions that correspond to a very small number of in-line-of-sight wind waves (0.02% of total waves on the most sheltered side of the embayment) (Extended Data Fig. 9) was sufficient to reasonable reproduce beach curvature.

In addition to the above, selecting an offshore location to represent the wave regime is a non-trivial issue that is often overlooked in shoreline studies. This is because it can significantly impact the statistics of the offshore directional spectra, which in turn affects the modelled shoreline solutions. The application of the BPM to Tróia-Sines (Extended Data Fig. 4) provides a good example of how well the model manages this issue. This embayment features a 50 km long N–S beach that faces sizable spatial changes in offshore mean wave power direction, with a shift of about 2.5° counterclockwise per each 1° increase in latitude (based on ERA5´s data^35^). The optimization function of the BPM reduces the impact of the spatial variability of the wave regime on the simulated shorelines by adjusting the local effects of shelter through relocating the UW fixed-point. A sensitivity analysis was conducted to assess the impact of the offshore wave data point location on the shoreline simulations using all ERA5’s wave data points within a geodesic distance of 1° from the originally selected offshore location. The analysis yielded comparable shorelines, with RMS errors from 52 to 102 m (average RMS of 71 m).

## The beach planform model: key characteristics, advantages and limitations

The Beach Planform Model (BPM) is a newly developed reduced-complexity model that can accurately describe equilibrium configuration of embayed beaches. This simplified approach is grounded on the assumption that embayed beach geometry is directly related to offshore wave sheltering. It assumes that, at each location, the shoreline aligns perpendicularly to the long-term mean wave power direction of in-line-of-sight offshore waves. In this sense, BPM depends solely on key morphological parameters and offshore oceanographic forcing, including the location of two fixed-point shelters, and a comprehensive characterization of the offshore wave climate, encompassing sea and swell directional spectra. Notably, BPM does not require a detailed description of bathymetry, and also does not involve any wave transformation processes, such as shoaling, refraction, diffraction and breaking.

In this model we tried to find an appropriate-complexity approach to describe the link between oceanographic forcing and morphological response. This approach places BPM between empirical models, which oversimplify this link, and more complex ones that require detailed system descriptions and processes parameterizations. These parameterizations can introduce imperfections that cascade up through scales of the model and compromise the reliability of the results^36^. By striking this balance, BPM distinguishes itself from both oversimplified and overly complex models.

At the present stage of development BPM builds the equilibrium planform of embayed beaches focusing solely on optimizing the upcoast headland. A drawback is that it may fall short in predicting the shape of highly indented pocket beaches, where both upcoast and downcoast headlands exert similar influences, or of beach areas dominated by wave diffraction. Similarly, BPM may not be able to account for the spatio-temporal variability imposed, for instance, by the wave climate seasonality or bathymetric irregularities such as canyon heads, islands or shoals. Addressing these limitations requires further research to enhance the model’s capabilities or consider the application of process-based models that can accommodate more complex and site-specific conditions^24,27,37^.

Notwithstanding the above limitations, BPM has demonstrated its efficacy in accurately describing embayed beach configurations across a diverse range of scenarios, encompassing both synthetic simulations and real-world environments. The curvature of the bay's equilibrium planform is observed to increases with the obliquity of the mean wave direction and decreases with the spread of wave directions. A substantial advantage of BPM is versatility and ease of application with minimal input requirements, making this simplified approach accessible to both researchers and practitioners. Moreover, the necessary information for running BPM (offshore waves^35,38–40^ and shoreline data^2,41,42^) is readily available on the internet, further streamlining its implementation.

Overall, our analysis reveals that the configuration of embayed beaches is primarily influenced by offshore wave sheltering. BPM application to equilibrium embayed beaches showed that at each location the shoreline aligns perpendicularly to the line-of-sight of mean offshore wave power direction. In general, using the long-term total sea state yielded accurate results. However, in embayments exposed to highly oblique offshore swells that are extremely narrow-banded, incorporating sea and swell partitioning can be a required procedure to simulate the shoreline curvature in the (otherwise) shadowed side of the embayment.

BPM provides unprecedented insights into how equilibrium embayed beach geometry is related to offshore wave climate and how it is modulated by sheltering. This allows for a deeper understanding of embayed beach dynamics and to explore potential impacts of climate change on their planform configuration, particularly in relation to changing wave patterns. The comprehensive analysis of embayed beach behaviour provided by BPM facilitates more accurate predictions, ultimately supporting better informed decisions regarding coastal management.

## Methods

### Beach planform model (BPM)

BPM is a Python-based model that predicts the equilibrium beach planform.

The model utilizes a finite-difference approach to build a shoreline that reflects the spatial variation of offshore wave sheltering along a beach limited by headlands. The BPM consists of two main components: the Shoreline Builder Function (SBF) and an optional Optimization Function (OF) (Extended Data Fig. 1). SBF requires the definition of an offshore wave climate, a beach start node (downwave beach limit), and the downwave (DW) and upwave (UW) headlands that bound the beach. The SBF then uses these inputs to create a shoreline that reflects the wave sheltering along the beach. The optional OF can be used to accommodate local effects of shelter by relocating the UW fixed-point, to the effective sheltering point, resulting in a better fit between the simulated and observed shorelines.

The iterative process of SBF starts by filtering the offshore waves at the beach start to keep only those within in-line-of-sight (disregarding all waves blocked as indicated in Extended Data Fig. 2). The mean (power) direction of the incident waves is then computed (see Methods—Wave power), and a shoreline segment perpendicular to that direction is added to that beach node. The endpoint of this segment becomes the starting node for the next iteration, and this process continues until the beach end is reached (Extended Data Figs. 1–2). At this point, if there is no need to optimize the position of the UW headland (the only free parameter of BPM), the model returns the simulated shoreline and the SBF is finalized. However, if optimization of UW is required, the BPM will use the OF to find its best-fitted position. OF utilizes the digitized UW headland position as an initial estimate and uses the Nelder-Mead optimization algorithm^43^ to resolve the ambiguity and determine the appropriate UW fixed-point location. This algorithm is chosen for its suitability in multidimensional unconstrained optimizations and robustness in handling small-amplitude noise The objective function in the optimization process aims to minimize the Root Mean Square (RMS) distance between SBF's output shoreline and the validation shoreline (Extended Data Fig. 1).

### Wave power

The wave power magnitude (P), for offshore wave timeseries and for incident waves at each node, was computed through the deep-water wave power formula^44^, Eq. (1) as

1 $$P = \frac{{\rho g^{2} Hm0^{2} Tm}}{64\pi }$$

where ρ is the density of seawater, g is the acceleration due to gravity, Hm0 is the significant wave height and Tm is the mean wave period. The mean power direction of waves was assessed through a power-weighted vector mean approach. The shelter wave power ratio (SWPR), at each shoreline node, was calculated by dividing the total wave power of the incident waves by the total offshore wave power.

### Data: idealized beach scenarios

The BPM model was utilized to reproduce the ensemble of idealized oblique wave conditions presented in Hurst et al.^24^ for the purpose of modelling bay equilibrium morphology under specific incident wave climates. The geomorphological parameters involve an embayment bounded at both ends by headland-fixed points, which represent either headlands or other rigid structures. The synthetic wave conditions consisted of ten thousand waves with constant wave height and wave period of 1 m and 6 s, respectively. Their directions were normally distributed, with standard deviation varying from ϴ_std_ = 5° to ϴ_std_ = 40° at five-degree increments while mean direction varied between ϴ_mean_ = − 5° and ϴ_mean_ =  + 60° at, similar intervals. These parameters matched the ones utilized by Hurst et al.^24^ in their research.

To address the limitations of the model for completely sheltered coastal areas, especially those that arose from narrow band waves, a solution was devised to expand the simulation coverage to the entire embayment. To achieve this, an extra low-energy and wide-banded background wave climate was introduced into the simulation. This additional synthetic wave climate had a constant height of 0.01 m, a period of 6 s, a broad directional spread angle of 45° and a mean direction equal to the other higher-energy components.

### Data: real beach cases

The BPM was applied to 13 embayed beaches worldwide covering a wide range of geomorphological and oceanographical settings (Figs. 2, 3, 4, Table 1 and Extended data Fig. 3). The headlands enclosing the embayed beach were visually located over Basemap imagery provided by ArcGIS Pro (sourced from Online Service ESRI via https://services.arcgisonline.com/arcgis/rest/services/World_Imagery/MapServer) and were used to define two fixed-points at the land-sea interfaces: a downwave headland (Dw), limiting the more exposed sector of the embayment, and an upwave headland (Uw) limiting the more sheltered sector of the embayment. The use of Uw and Dw terminology, which pertains to wave propagation directions and their impact on sheltering, is preferred over terminologies associated with longshore sediment transport directions (updrift and downdrift), generally used in coastal studies^7^. This is due to the occasional lack of alignment between the two terminologies as evidenced by Tróia-Sine’s embayment, where the Uw headland is located at the downdrift end of the beach^45,46^ and because it highlights the relevance of wave sheltering effects as opposed to sediment transport processes.

Beach start was defined by the xy coordinates of the shoreline's downwave end, specifically in the area that is more exposed within the embayment. This location marks where a rocky coast transitions into sand and becomes a beach.

The validation shorelines correspond to time-average conditions. These shorelines were obtained by vectorizing the limit between water and sand on multi-year (approx. 7.5 years) averaged Sentinel-2 Level 2A image layers provided by ArcGIS PRO (sourced from the Planetary Computer Sentinel-2 Level2A data catalog on Azure via https://sentinel.imagery1.arcgis.com/arcgis/services/Sentinel2L2A/ImageServer). At each embayed beach, the average image used is the overlapping pixel values from all available sentinel-2-Level 2A images at the site, from mid-2015 to 2022 (encompassing several hundreds of images). Additionally, to improve water–sand limit recognition, we have rendered an average of all available sentinel-2 images into a colour-infrared visualization using Near Infrared, Red and Blue in RGB bands, respectively. For consistency purposes, all shorelines were projected either in Universal Transverse Mercator (UTM) or in Transverse Mercator (TM) projections. The assessment of the fitting between simulated and validation shorelines was done by computing the RMS error using the coastline builder (refer to BPM description in the Methods). To normalize this error, we divided it by the shoreline length, resulting in NRMS.

The offshore wave regime used in real beach cases was obtained from the European Centre for Medium-Range Weather Forecast’s ERA5 reanalysis of the global climate^35^. This dataset includes ocean-wave information on a regular lat.-long. grid of 0.5˚. Here we used ERA5 data at 3-h intervals for the period between 01 Jan 1979 00:00 and 31 Dec 2021 21:00. Simulations with total sea state used significant height of combined wind waves and swell (SWH), mean wave direction (MWD) and mean wave period (MWP). Simulations with sea and swell partitions, utilized significant height, mean direction and mean period of sea (SHWW, MDWW and MPWW) and total swell partitions (SHTS, MDTS and MPTS), respectively.

### Supplementary Information

Supplementary Information 1.

Supplementary Information 2.

Supplementary Information 3.

Supplementary Information 4.

Supplementary Information 5.

Supplementary Information 6.

Supplementary Information 7.

Supplementary Information 8.

Supplementary Information 9.

Supplementary Information 10.

## Data Availability

The ERA5 data described in Hersbach et al.^35^ was downloaded from the Copernicus Climate Service (https://cds.climate.copernicus.eu/cdsapp#!/dataset/reanalysis-era5-single-levels).

## Code availability

The Python codes are made available in GitHub repository https://github.com/AnaNobre/BeachPlanformModel.git.

## References

[1] Vousdoukas MI (2020). Sandy coastlines under threat of erosion. Nat. Clim. Chang..

[2] Luijendijk A (2018). The state of the world’s beaches. Sci. Rep..

[3] Mentaschi L, Vousdoukas MI, Pekel J-F, Voukouvalas E, Feyen L (2018). Global long-term observations of coastal erosion and accretion. Sci. Rep..

[4] Inman DL, Nordstrom CE (1971). On the tectonic and morphologic classification of coasts. J. Geol..

[5] Castelle B, Robinet A, Idier D, D’Anna M (2020). Modelling of embayed beach equilibrium planform and rotation signal. Geomorphology.

[6] Hsu JR-C (2008). Appreciation of static bay beach concept for coastal management and protection. J. Coast. Res..

[7] Hsu JR-C, Yu M-J, Lee F-C, Benedet L (2010). Static bay beach concept for scientists and engineers: A review. Coast. Eng..

[8] Klein AHF (2010). Morphodynamics of structurally controlled headland-bay beaches in southeastern Brazil: A review. Coast. Eng..

[9] Hsu JRC, Takaaki UB, Richard S (1993). Beaches downcoast of harbours in bays. Coast. Eng..

[10] Silvester R (1960). Stabilization of sedimentary coastlines. Nature.

[11] Kemp J, Vandeputte B, Eccleshall T, Simons R, Troch P (2018). A modified hyperbolic tangent equation to determine equilibrium shape of headland bay beaches. Coast. Eng. Proc..

[12] Tran YH, Barthélemy E (2020). Combined longshore and cross-shore shoreline model for closed embayed beaches. Coast. Eng..

[13] Simmons, J. A., Splinter, K. D., & Beuzen, T. Data-driven modelling of shoreline evolution, in *Coastal Sediments 2019—Proceedings of The 9th International Conference* (eds. Wang, P., Rosati, J. D. & Vallee, M.) (World Scientific, 2019).

[14] Krumbein, W. C. *Shore Processes and Beach Characteristics*. vol. 3 (U.S. Beach Erosion Board, 1947).

[15] Yasso WE (1965). Plan geometry of headland-bay beaches. J. Geol..

[16] Mashima Y (1961). Stable configuration of coast line. Coast. Eng. Jpn..

[17] Hsu JRC, Evans C (1989). Parabolic bay shapes and applications. Proc. Inst. Civ. Eng..

[18] Hsu John RC, Richard S, Xia Y-M (1989). Static equilibrium bays: New relationships. J. Waterway Port Coast. Ocean Eng..

[19] Tan SK, Chiew YM (1994). Analysis of bayed beaches in static equilibrium. J. Waterway Port Coast. Ocean Eng..

[20] Elshinnawy AI, Medina R, González M (2018). Dynamic equilibrium planform of embayed beaches: Part 1. A new model and its verification. Coast. Eng..

[21] Jaramillo C, Jara MS, González M, Medina R (2021). A shoreline evolution model for embayed beaches based on cross-shore, planform and rotation equilibrium models. Coast. Eng..

[22] González M, Medina R (2001). On the application of static equilibrium bay formulations to natural and man-made beaches. Coast. Eng..

[23] Moreno, L. J. & Kraus, N. C. Equilibrium shape of headland-bay beaches for engineering design, in *Proceedings, Coastal Sediments 1999* 860–875 (American Society of Civil Engineers, 1999).

[24] Hurst MD, Barkwith A, Ellis MA, Thomas CW, Murray AB (2015). Exploring the sensitivities of crenulate bay shorelines to wave climates using a new vector-based one-line model. J. Geophys. Res. Earth Surf..

[25] Hanson, H. & Kraus, N. C. *GENESIS: Generalized model for simulating shoreline change, Report 1 technical reference* (US Army Corps of Engineers Waterways Experiment Station, 1989).

[26] Weesakul S, Rasmeemasmuang T, Tasaduak S, Thaicharoen C (2010). Numerical modeling of crenulate bay shapes. Coast. Eng..

[27] Robinet A, Idier D, Castelle B, Marieu V (2018). A reduced-complexity shoreline change model combining longshore and cross-shore processes: The LX-Shore model. Environ. Model. Softw..

[28] Buccino M, Tuozzo S, Ciccaglione MC, Calabrese M (2021). Predicting crenulate bay profiles from wave fronts: Numerical experiments and empirical formulae. Geosci. J..

[29] French J (2016). Appropriate complexity for the prediction of coastal and estuarine geomorphic behaviour at decadal to centennial scales. Geomorphology.

[30] Ashton A, Murray AB, Arnoult O (2001). Formation of coastline features by large-scale instabilities induced by high-angle waves. Nature.

[31] Short AD (2010). Role of geological inheritance in Australian beach morphodynamics. Coast. Eng..

[32] Lausman R, Klein AHF, Stive MJF (2010). Uncertainty in the application of the Parabolic Bay Shape Equation: Part 1. Coast. Eng..

[33] Robinet A, Castelle B, Idier D, Harley R, Splinter KD (2020). Controls of local geology and cross-shore/longshore processes on embayed beach shoreline variability. Mar. Geol..

[34] Speranski N, Calliari L (2001). Bathymetric lenses and localized coastal erosion in Southern Brazil. J. Coast. Res..

[35] Hersbach, H., Bell, B., Berrisford, P., Biavati, G., Horányi, A., Muñoz Sabater, J., Nicolas, J., Peubey, C., Radu, R., Rozum, I., Schepers, D., Simmons, A., Soci, C., Dee, D., Thépaut, J.-N. ERA5 Hourly Data on Single Levels from 1979 to Present; Copernicus Climate Change Service (C3S) Climate Data Store (CDS). Accessed 17 Jan 2023. 10.24381/cds.adbb2d47.

[36] Murray AB, Shroder JF (2013). 2.5 Which models are good (enough), and when?. Treatise on Geomorphology.

[37] Gainza J, González EM, Medina R (2018). A process based shape equation for a static equilibrium beach planform. Coast. Eng..

[38] Reguero BG, Menéndez M, Méndez FJ, Mínguez R, Losada IJ (2012). A Global Ocean Wave (GOW) calibrated reanalysis from 1948 onwards. Coast. Eng..

[39] Tolman, H. L. User manual and system documentation of WAVEWATCH III TM version 3.14 y. Technical Note, (MMAB Contribution, 276(220), 2009).

[40] Jiang X, Xie B, Bao Y, Song Z (2023). Global 3-hourly wind-wave and swell data for wave climate and wave energy resource research from 1950 to 2100. Sci Data.

[41] Vos K, Splinter KD, Harley MD, Simmons JA, Turner IL (2019). CoastSat: A Google Earth Engine-enabled Python toolkit to extract shorelines from publicly available satellite imagery. Environ. Model. Softw..

[42] Gorelick N (2017). Google Earth Engine: Planetary-scale geospatial analysis for everyone. Remote Sens. Environ..

[43] Nelder JA, Mead R (1965). A simplex method for function minimization. Comput. J..

[44] Bretschneider, C. L. & Cengiz Ertekin, R. Estimation of wave power as an energy resource for Hawaii, in *Ocean Energy Recovery* 189–201 (American Society of Civil Engineers, 1990).

[45] Gama, C., Taborda, R. & Andrade, C. Longshore sediment transport in the Tróia-Sines Littoral Ribbon (SW Portugal), in *VII Congresso Nacional de Geologia, Vol. II* 389–392 (Universidade de Évora, 2006).

[46] Rebêlo L, Ferraz M, Brito P, Terrinha P (2012). Quantification of sediments accumulated in the NW sector of Tróia Peninsula (Portugal) between 1928 and 1995. J. Coast. Conserv..

