# Supplementary material for: Embayed beach configuration explained by wave sheltering
Source: Sci Rep. 2024 Jan 11;14:1099. doi: 10.1038/s41598-024-51574-x (PMC10784308; doi:10.1038/s41598-024-51574-x)
Supplement: Supplementary file 10 — Supplementary Information 10. [file 41598_2024_51574_MOESM10_ESM.docx]

# Supplementary Material – extended data figure legends

Extended Data Figure 1 | Flowchart showing the BPM's steps for shoreline simulation. BPM includes two model functions, the main Shoreline Builder Function (SBF) and the optional Optimization Function (OF). The model inputs are represented in light green boxes and output is represented in the light blue box. The SBF’s iterative process of offshore wave filtering, at each shoreline node, is illustrated in the right side of the panel. DW and UW are headlands positioned at the embayment's downwave and upwave ends (see Methods –Beach Planform Model for details).

Extended Data Figure 2 | Beach Planform Model. (a) Schematic representation of the BPM offshore wave filtering along an idealized embayment. DW and UW are headlands (fixed-points) positioned at the embayment's downwave and upwave ends (see Online Methods – Beach Planform Model for details). b, c and d showcase the wave filtering process at three shoreline nodes with decreasing sheltering from b to d. Incident (blocked) waves are those in-line-of-sight (out-of-sight) at each node. The size and direction of the black arrows reflect the mean wave power magnitude and direction. Map created in ArcGIS Pro 3.2.0, <https://www.esri.com/en-us/arcgis/products/arcgis-pro> .

Extended Data Figure 3 | ERA5’s long-term wave height and directions at the study sites. The wave rose diagrams reflect the significant height of combined wind waves and swell (SWH) and mean wave direction (MWD), at each study site identified by their latitude and longitude coordinates. The Agraria and Paraíso study sites share the same ERA5 location owing to their proximity. The coloured lines represent the best shoreline solution, at each beach case, labelled by shelter wave power ratio (SWPR) along the embayment (solid-green represents simulations with total sea state and blue-dashed represents simulations with sea and swell partitions).

Extended Data Figure 4 | BPM’s optimized shorelines, shelter wave power ratio and normalized root mean square (NRMS) error at Tróia-Sines (Portugal), Narrabeen and Cooloola (Australia) embayments. The left and central panels show simulations using total sea state and sea and swell partitions, respectively. The right panels show both BPM’s simulations (green-solid and blue-dashed lines) overlayed on validation (satellite-derived) shoreline (red-dashed lines). The coloured arrows represent the incident (in-line-of-sight) wave climate, with arrow size and colour indicating magnitude, and arrow direction indicating mean wave power direction along the embayment. Map created in ArcGIS Pro 3.2.0, basemap imagery source is Esri’s Earthstar geographic, <https://www.esri.com/en-us/arcgis/products/arcgis-pro> .

Extended Data Figure 5 | BPM’s optimized shorelines, shelter wave power ratio and normalized root mean square (NRMS) error at Florianópolis and Ponta Negra (Brazil) embayments. The left and central panels show simulations using total sea state and sea and swell partitions, respectively. The right panels show both BPM’s simulations (green-solid and blue-dashed lines) overlayed on validation (satellite-derived) shoreline (red-dashed lines). The coloured arrows represent the incident (in-line-of-sight) wave climate, with arrow size and colour indicating magnitude, and arrow direction indicating mean wave power direction along the embayment. Map created in ArcGIS Pro 3.2.0, basemap imagery source is Esri’s Earthstar geographic, https://www.esri.com/en-us/arcgis/products/arcgis-pro.

Extended Data Figure 6 | BPM’s optimized shorelines, shelter wave power ratio and normalized root mean square (NRMS) error at Boggoms and Gamtoos river mouth (South Africa) embayments. The left and central panels show simulations using total sea state and sea and swell partitions, respectively. The right panels show both BPM’s simulations (green-solid and blue-dashed lines) overlayed on validation (satellite-derived) shoreline (red-dashed lines). The coloured arrows represent the incident (in-line-of-sight) wave climate, with arrow size and colour indicating magnitude, and arrow direction indicating mean wave power direction along the embayment. Map created in ArcGIS Pro 3.2.0, basemap imagery source is Esri’s Earthstar geographic, <https://www.esri.com/en-us/arcgis/products/arcgis-pro> .

Extended Data Figure 7 | BPM’s optimized shorelines, shelter wave power ratio and normalized root mean square (NRMS) error at Pismo (USA), Family Jiménez and Marron (Mexico) embayments. The left and central panels show simulations using total sea state and sea and swell partitions, respectively. The right panels show both BPM’s simulations (green-solid and blue-dashed lines) overlayed on validation (satellite-derived) shoreline (red-dashed lines). The coloured arrows represent the incident (in-line-of-sight) wave climate, with arrow size and colour indicating magnitude, and arrow direction indicating mean wave power direction along the embayment. The lower-left panel illustrates the existence of multiple protruding headlands and includes an extent indicator of the lower-central panel. Map created in ArcGIS Pro 3.2.0, basemap imagery source is Esri’s Earthstar geographic, <https://www.esri.com/en-us/arcgis/products/arcgis-pro>.

Extended Data Figure 8 | BPM’s optimized shorelines, shelter wave power ratio and normalized root mean square (NRMS) error at Puerto Huarmey, Agraria and Paraíso (Peru) embayments. The left and central panels show simulations using total sea state and sea and swell partitions, respectively. The right panels show both BPM’s simulations (green-solid and blue-dashed lines) overlayed on validation (satellite-derived) shoreline (red-dashed lines). The coloured arrows represent the incident (in-line-of-sight) wave climate, with arrow size and colour indicating magnitude, and arrow direction indicating mean wave power direction along the embayment. In the lower-left panel, Paraíso, the displacement of the fitted UW was too large to fit properly. Map created in ArcGIS Pro 3.2.0, basemap imagery source is Esri’s Earthstar geographic, <https://www.esri.com/en-us/arcgis/products/arcgis-pro>.

Extended Data Figure 9 | ERA5’s long-term wave roses at Paraíso. The lines correspond to validation (red-dashed) and simulated (green-solid and blue-dashed) shorelines labelled by shelter wave power ratio (SWPR). Panel (a) depicts the wave rose of significant height of combined wind waves and swell (SWH) and mean wave direction (MWD) in total sea state simulations, with no records between 270-360° directions in the respective dataset. Panel (b) displays the joint partitions of sea and swell, with sea wave partitions (SHWW and MDWW) and total swell partitions (SHTS and MDTS), with 74 records between 270-360°, which account for only 0.003% of the total records. Right wave roses represent the distribution of records up to 0.05% of the data, in their respective wave dataset.
